# Complementarity among natural enemies enhances pest suppression

**DOI:** 10.1038/s41598-017-08316-z

**Published:** 2017-08-15

**Authors:** Matteo Dainese, Gudrun Schneider, Jochen Krauss, Ingolf Steffan-Dewenter

**Affiliations:** Department of Animal Ecology and Tropical Biology, Biocenter, University of Würzburg, Am Hubland, 97074 Würzburg Germany

## Abstract

Natural enemies have been shown to be effective agents for controlling insect pests in crops. However, it remains unclear how different natural enemy guilds contribute to the regulation of pests and how this might be modulated by landscape context. In a field exclusion experiment in oilseed rape (OSR), we found that parasitoids and ground-dwelling predators acted in a complementary way to suppress pollen beetles, suggesting that pest control by multiple enemies attacking a pest during different periods of its occurrence in the field improves biological control efficacy. The density of pollen beetle significantly decreased with an increased proportion of non-crop habitats in the landscape. Parasitism had a strong effect on pollen beetle numbers in landscapes with a low or intermediate proportion of non-crop habitats, but not in complex landscapes. Our results underline the importance of different natural enemy guilds to pest regulation in crops, and demonstrate how biological control can be strengthened by complementarity among natural enemies. The optimization of natural pest control by adoption of specific management practices at local and landscape scales, such as establishing non-crop areas, low-impact tillage, and temporal crop rotation, could significantly reduce dependence on pesticides and foster yield stability through ecological intensification in agriculture.

## Introduction

Globally, numerous pests attack crops causing large economic damage without efficient conventional or biological control^1^. Importantly, the control of pests through the application of chemical pesticides repeatedly fails to provide reliable conventional control and contributes to significant environmental harm^2^. For instance, the overuse of pesticides can lead to pest resurgence^3, 4^, as well as population decline of beneficial insects like natural enemies^2^ and pollinators^5, 6^. Therefore, there is an increasing interest in approaches that reconcile high crop yields with environmental sustainability through ecological intensification and the targeted management of ecosystem services in agriculture^7, 8^.

Pest control by natural enemies arises as an ecologically and economically promising solution^9^. Natural enemies have been estimated to account for at least 50% of pest control occurring in crop fields^2^ providing an essential ecosystem service valued at $13 billion per year in the USA alone^10^. Among natural enemies, both predatory and parasitic insects have been shown to be effective in suppressing pest species^11, 12^. Because these species coexist in natural communities, they are potentially involved in positive or negative interactions with each other that may influence the strength of pest regulation. In fact, empirical studies indicate that trophic interactions among diverse natural enemy assemblages may result in a full spectrum of outcomes including null, additive, antagonistic or synergistic effects^13^. For instance, parasitoids and predators can attack a pest during different periods of its occurrence in the field^14^, resulting in stronger pest suppression than a single-enemy species^15^. However, this additive effect may be diminished by antagonistic interactions between natural enemies such as intraguild predation^16^. This can occur when, for example, predators eat immature parasitoids within their prey, thus reducing parasitoid impact on the pest^17^. However, exactly how these positive and negative interactions among predators and parasitoids affect natural pest control often remains an open question. Many studies consist of surveys that sample only one period of the pest occurrence in the field, or focus only on one enemy, making it difficult to understand the combined effects of complete natural enemy guilds on pest population dynamics.

The landscape in which a field is embedded can also play a fundamental role in shaping the natural enemy community^18^, and consequently the delivery of natural pest control services^16, 19, 20^. Complex landscapes with a high density of uncultivated and perennial habitats are often found to enhance the natural enemy populations and support better biological control^12, 21^. In addition, complex landscapes can potentially reduce antagonistic interactions among insect natural enemies, thereby allowing coexistence of species with overlapping functional niches^22^. However, studies are lacking that examine the combined contribution of natural enemy guilds to pest control across different periods of the pest occurrence in the field while addressing landscape scale effects in parallel.

We used a field exclusion experiment, to examine the degree to which different functional guilds of natural enemies can reduce a pest population. We quantified the combined effects of parasitoids and ground-dwelling predators on the population dynamics of pollen beetles in 18 oilseed rape (OSR) fields while simultaneously contrasting the landscape context of the fields (Supplementary Fig. S1). The pollen beetle, *Meligethes aeneus* F., (Coleoptera: Nitidulidae), is one of the major pests of OSR in Europe that cause significant economic damage and lacks efficient conventional or biological control^23, 24^. To date, most previous studies have focused on parasitoids that attack pollen beetle larvae during the flowering stage^12, 25, 26^. Ground-dwelling predators may also exert a considerable pollen beetle mortality in OSR^27, 28^ as they attack mature larvae when they drop to the soil to pupate, but their actual predation rates have not been assessed. In this context, OSR provides an interesting experimental case to better comprehend the strength of pest control exerted by different natural enemies acting on different periods of the pest occurrence in the field. Specifically, we aimed (*i*) to quantify the individual and combined effects of ground-dwelling predators and parasitoids on controlling pollen beetle densities in OSR, and (*ii*) to assess whether biological control efficacy is affected by the surrounding landscape.

## Results

### Impact of natural enemies on pest density

**Figure 1:**
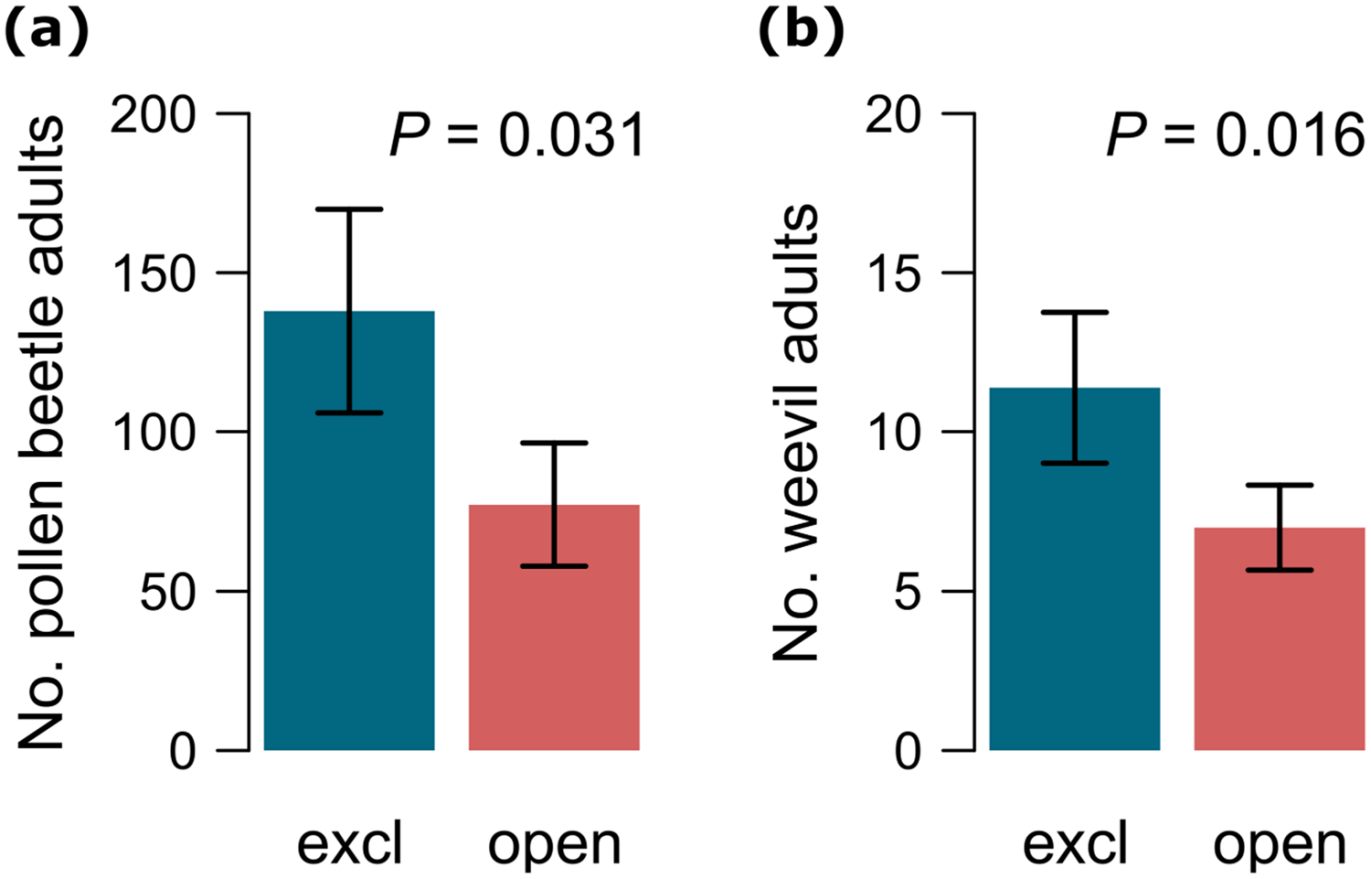
Effect of ground-dwelling predator exclusion (excl = exclosure treatment; open = open treatment) on pest density. Mean ( ± SE) (**a**) number of adult pollen beetles (*Meligethes aeneus*) and (**b**) number of adult OSR pest weevils (*Ceutorhynchus* sp.).

In the exclusion experiment, in the absence of ground-dwelling predators the densities of adult pollen beetles were significantly higher than in the open control treatments (linear mixed effect models, LMMs: *F*_1,17_ = 5.56, *P* = 0.031) (Table 1). The average number of pollen beetle adults was reduced by 44% in the open compared to the exclosure treatment (Fig. 1a). Further, the average number of emerged OSR pest weevils (*Ceutorhynchus* sp.) was reduced by 38% in the open treatment compared to the exclosure treatment (LMMs: *F*_1,17_ = 7.14, *P* = 0.016) (Fig. 1b).

**Table 1:** Results of linear mixed effects models (LMMs) relating pest density and parasitism to explanatory variables. Only significant main effects and interactions are shown, after backward selection procedure (% Non-crop habitat was retained in the model for pollen beetle adult despite having a high *P*-value because the interaction with parasitism remained significant).

Explanatory variables	dDF	*F*-value	*P*-value
**Pest density**			
*Pollen beetle larvae*			
% Non-crop habitat	67	12.675	0.001
*Pollen beetle adult*			
Treatment	17	5.58	0.031
Parasitism	14	10.08	0.007
% Non-crop habitat	14	2.15	0.165
Parasitism × % Non-crop habitat	14	5.08	0.041
**Parasitism**			
*Proportion of pollen beetle parasitized*			
Abundance pollen beetle larvae	68.53	5.75	0.019
% Grasslands	66.15	6.78	0.011

**Figure 2:**
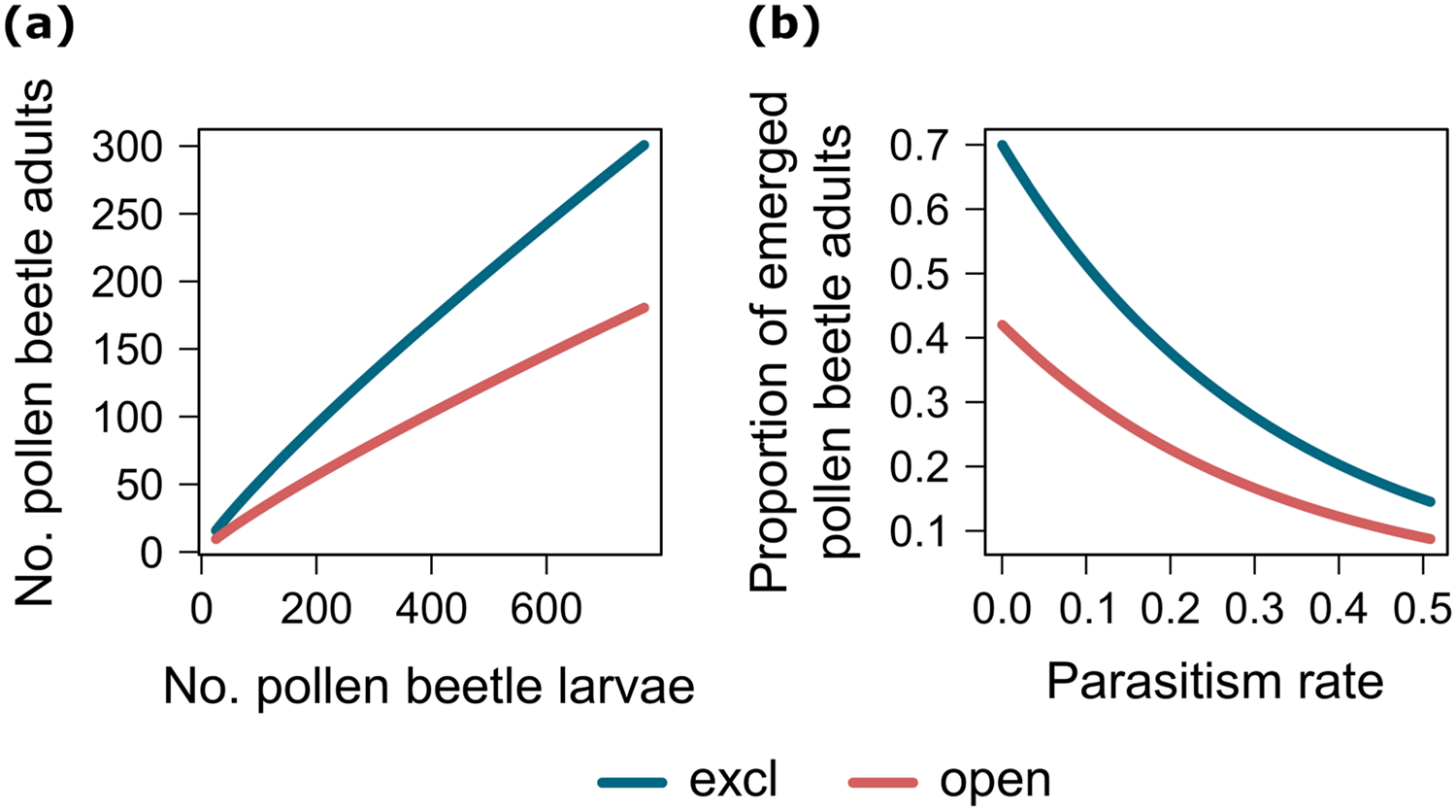
(**a**) Relationship between number of adult pollen beetles emerging from the soil and number of pollen beetle larvae dropping to the ground. (**b**) Effect of parasitism on the proportion of adult pollen beetles emerging from the soil. Separate relationships were reported for the two exclusion treatments (excl = exclosure treatment; open = open treatment). The interaction with treatment was not significant (*P* > 0.05) in both models. Fitted lines are back-transformed linear mixed model estimates from the model described in Table 1 (figures on ln-transformed scale are provided in Supplementary Fig. S1).

The density of pollen beetle adults emerging from the ground was positively correlated with the density of pollen beetle larvae dropping to the ground (LMMs: *F*_1,16_ = 16.80, *P* =  < 0.001) (Fig. 2a; Supplementary Fig. S2a). Considering the proportion of emerged pollen beetles, there was an additive effect between the proportion parasitized (LMMs: *F*_1,16_ = 7.24, *P* = 0.016) and the ‘exclusion’ treatment (LMMs: *F*_1,16_ = 5.56, *P* = 0.031) (Table 1). Parasitism also correlated negatively with the proportion of pollen beetles emerging in both exclosure and the open treatments (Fig. 2b; Supplementary Fig. S2b).

### Interaction with landscape context

**Figure 3:**
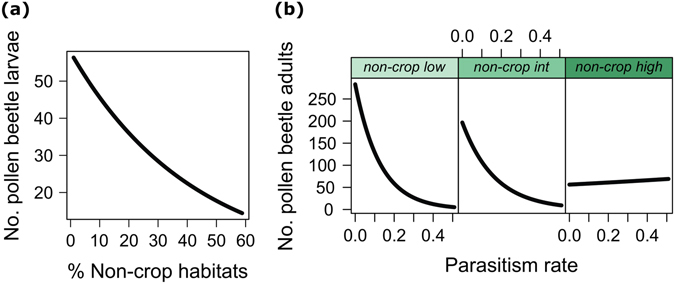
Effect of landscape context on pest density. (**a**) Effect of the proportion of non-crop habitats in the landscape on the number of pollen beetle larvae dropping to the ground. (**b**) Interactive effect of parasitism rate and the proportion of non-crop habitats in the landscape on the number of adult pollen beetle emerging from the soil; panels are ranked from left to right according to increasing proportion of non-crop habitats (*non-crop low*, landscapes with low cover of non-crop habitats −6%; *non-crop int*, landscapes with intermediate cover of non-crop habitats −18%; *non-crop high*, landscapes with high cover of non-crop habitats −50%). Fitted lines are back-transformed linear mixed model estimates from the model described in Table 1 (Supplementary Fig. S4).

An increased proportion of non-crop habitats in the landscape was associated with a significantly decreased density of pollen beetle larvae (Table 1 and Fig. 3a; Supplementary Fig. S4a). Conversely, no significant effect of the proportion of non-crop habitats in the landscape was found for densities of ground-dwelling predators (total, ground beetles, rove beetles and spiders) or proportion of beetles parasitized (LMMs, *P* > 0.05). Considering the main habitat types separately, the proportion of grasslands in the landscape and the density of pollen beetle larvae were positively correlated with the proportion of parasitized larvae (Table 1; Supplementary Fig. S3b).

There was a significant interaction between the proportion of non-crop habitats in the landscape and the proportion of beetle larvae parasitized for the density of emerged pollen beetle adults (Table 1). The density of emerged pollen beetles was negatively correlated with parasitism in landscapes with a low or intermediate proportion of non-crop habitats, but disappeared in landscapes with a high proportion of non-crop habitats (Fig. 3; Supplementary Fig. S4b).

## Discussion

Our study explored the combined effect of parasitoids and ground-dwelling predators on the biological control of pollen beetles in oilseed rape fields (OSR). Combining an exclusion experiment with pollen beetle sampling over two life stages allowed us to directly assess the importance to pest control of two different natural enemy guilds and the surrounding landscape. Our results suggest temporal complementarity among natural enemies in controlling pollen beetle populations, likely due to parasitoids and ground-dwelling predators attacking different periods of the pest occurrence in the field. We demonstrated that generalist predators exerted a considerable pest control and, complemented specialist parasitoids. We also found that such benefits improved when an increasing proportions of the surrounding landscape was occupied by non-crop habitats. Although this study covered only one crop season, our results highlight the importance of contributions from different natural enemy guilds to pest regulation in crops. However, it would be interesting to verify these results over a longer temporal scale.

The observed reductions in pollen beetle adults (44%) and weevils (38%) due to ground-dwelling predators were agriculturally significant given that a reduction of 30% has been identified as a threshold for effective biological control of pollen beetles^29^. Ground-dwelling predators, which prey on pollen beetle larvae as they drop to the soil to pupate, thus play a key role in reducing the density of adult beetles before they can pupate and emerge in summer. Whereas the impact of ground-dwelling predators on cereal aphids is usually small compared to other natural enemies^30–32^, their impact appears in OSR strong enough to control both pollen beetles and weevils. Our results for weevils are in agreement with a previous study^28^, which reported ground-dwelling predators as playing a key role in controlling stem weevils in OSR. However, conventional pest management strategies can negatively affect predator populations, thereby reducing the efficacy of natural pest control^33^. Alternative farming practices, such as organic farming^34^ or conservation tillage^35^, can potentially counteract these effects by sustaining more abundant populations of predators compared to intensively managed farming systems^36, 37^. In addition to the effect of ground-dwelling predators, we also found a significant effect of parasitism on pollen beetles; even though parasitism averaging 15.1% ± 0.03 SE remains below the threshold value required for effective biological control^29^, parasitoids did significantly reduce the density of emerging pollen beetles.

Significant negative interactions between natural enemies, such as intraguild predation of parasitized pollen beetle larvae by ground-dwellers, were not indicated in this study. Overall, parasitoids and predators had an additive impact on pest suppression in OSR, suggesting niche partitioning among parasitoids and ground-dwelling predators, likely as a result of them consuming a different developmental stage of the pest, support higher biological control^38^. Adding a life history-related temporal dimension to biological control will allow us to better comprehend the flow and stability of pest control services in agro-ecosystems^39^.

Landscape scale responses of pollen beetle densities and parasitism confirmed that non-crop habitats can be an important factor reducing pest pressure^12, 21^. The density of pollen beetle larvae in OSR fields decreased with an increasing proportion of non-crop habitats in the landscape. Previous studies have reported both a significant reduction of pollen beetle pressure^12, 21^ and a significant increase in pest pressure^40, 41^ with an increasing proportion of non-crop habitats in the landscape. Our finding is consistent with the hypothesis that complex landscapes containing a high proportion of non-crop habitats tend to reduce the density of pest populations compared to simple landscapes dominated by arable lands, due to better top-down control by natural enemies^18, 21^. This is confirmed by the positive correlation between parasitism rate and the proportion of grasslands in the landscape. Parasitoids benefit from the presence of uncultivated, perennial habitats that provide nectar resources, alternative hosts, and overwintering sites^18, 42^. Therefore, perennial habitats, such as grasslands, can enhance the number and life span of parasitoids that migrate into neighbouring crop fields and contribute to the reduction of pests^12^. However, such effects cannot be generalised to all natural enemies. For instance, we found that ground-dwelling predators were not influenced by landscape context. As other authors have noted^43–45^, not all natural enemy populations benefit from the availability of perennial habitats in the landscape because responses are species specific. Further studies focusing on the trait-specificity of landscape effects, for example, the role of feeding preferences, body size or dispersal capacity, might help to disentangle the different responses of natural enemies to landscape context^46, 47^.

Also, our results showed a strong effect of parasitism on pollen beetle control in landscapes with low or intermediate proportion of non-crop habitats, but not in landscapes with a high proportion of non-crop habitats. This result may reflect the density-dependent relationship between host and parasitoid. In accordance with a recent review^48^, we found parasitism to be positively density dependent, i.e. parasitism increased with increasing density of pollen beetle larvae. This density-dependent parasitism, together with the lower density of pollen beetle larvae in landscapes with a high proportion of non-crop habitats could explain the neutral effect of parasitism on pollen beetle control in complex landscapes with a high density of perennial habitats.

Overall, our results underline the complementary impact of different natural enemy guilds in regulating pest population in crops. Both parasitoids and ground-dwelling predators are important biocontrol agents acting on different periods of the pest occurrence in the field. Landscapes with a large area in non-crop habitats support natural pest control with abundant suitable habitats for natural enemies, whereas landscapes dominated by arable land would benefit from the adoption of specific management practices that mitigate the negative effects of landscape simplification^37, 49, 50^. For example, the implementation of flower strips or hedgerows in crop fields could provide additional habitats for different natural enemy guilds^49–52^. Preservation of different natural enemy guilds may also become increasingly important as an insurance policy against potential new pest problems arising from climate change^53^. Different species will not respond equally to climate change and temporal complementarity among natural enemies might prove to be an important mechanism for ensuring stable pest control. In conclusion, optimizing natural pest control could significantly reduce the dependence of modern agriculture on pesticide applications while maintaining high crop yields^7, 54^. A better knowledge of the direct connections between alternative pest control strategies, crop damage reduction and yield benefits is needed to demonstrate the profitability of wildlife-friendly farming practices.

## Methods

### Study area

Field experiments were conducted between April and June 2012. Eighteen conventionally managed OSR fields were selected in the surrounding of the city of Würzburg, Bavaria, Germany. The studied field sites were at least 2 km apart from each other and had an average field size of 2.0 ha ± 1.1 ha SD. We determined the proportion of OSR and the proportion of non-crop habitats (all uncultivated and perennial habitats such as forests and grasslands) in 1 km radius around each field using data from the ‘Bayerische Landesanstalt für Landwirtschaft (LfL)’ (Freising, Germany) in the software ArcMap (ESRI 2011. ArcGIS Desktop: Release 10. Redlands, CA, USA: Environmental System Research Institute, USA). In the study area, the proportion of OSR covered a range from 0.9% to 21.0% (mean ± SD = 7.4 ± 5.9) and the proportion of non-crop habitats varied from 1.0% to 58.8% (mean ± SD = 25.4 ± 17.0) (see Dataset S1). Among non-crop habitats, the proportion of forests covered a range from 0.7% to 48.2% (mean ± SD = 20.6 ± 14.8), while the proportion of grasslands varied from 0.2% to 17.3% (mean ± SD = 4.8 ± 5.2) (see Dataset S1). In each field, all measurements were done at a distance of about 3 m from the field edge, as we expected more pronounced effects at the field edge compared to the field centre^26^.

### Study organism

The oilseed rape pollen beetle *Meligethes aeneus* F. produces one generation per year, and adults overwinter predominantly in field margins, hedgerows and woodlands. In spring, when the temperature exceeds 10 °C, they emerge and start to feed on the pollen of various plants. As temperatures rise, adults disperse into winter OSR fields where they feed on and oviposit in the flower buds. The larvae feed on pollen and, when mature, drop to the soil for pupation. The next generation of adult beetles emerges during the summer, and these will overwinter^55^. Natural enemies comprise generalist ground-dwelling predators such as ground beetles, spiders and rove beetles, and specialised parasitic hymenoptera. The eggs or larvae of pollen beetle in Europe are parasitized by several species and among these *Tersilochus heterocerus* is one of the most important parasitoids specialized on pollen beetles in the study region^56^. *T. heterocerus* is an ichneumonid larval endoparasitoid and the females mainly oviposit in large, second-instar pollen beetle larvae in open flowers. After the pollen beetle larvae drop to the ground, *T. heterocerus* completes its development and kills the pre-pupal stage of its host^56^. Pollen beetles are most vulnerable to predation when, as mature larvae, they drop to the soil to pupate, but predation rates are currently unknown.

### Parasitism and predation

In each field, the contribution to natural pest control by ground-dwelling predators was quantified by establishing an exclusion experiment. Two treatments were used as follows: (*i*) an exclosure treatment that consisted of a metal ring (1 m diameter, 15 cm high) dug a few centimetres into the soil and (*ii*) an open treatment separated by 20 m from the exclosure treatment. A pitfall trap was placed in the middle of the exclosure treatment to trap accidentally enclosed predators. The experiment was set up at the beginning of April before oilseed rape started to flower.

We measured the density of pollen beetle larvae dropping to the ground with white plastic trays (surface area 656.25 cm²) filled with water and detergent that were placed under the OSR plants on the floor of the fields. The plastic trays were set up during OSR flowering on the 4^th^ of May and emptied weekly for four weeks. We determined parasitism rates by *T. heterocerus* by dissecting all pollen beetle larvae caught with plastic trays under a binocular and counting the black eggs of *T. heterocerus*.

For each treatment (exclosure and open), the number of pollen beetle adults emerging from the ground was surveyed with a photoeclector trap (0.56 m diameter) placed after OSR flowering (usually at the end of May, but the exact date differed between the field sites). In the open treatment, we put a pitfall trap inside the photoeclector to capture the ground-dwelling predators. In which way, we maintained comparable conditions between the two treatments during the survey of pollen beetle adult. The trapping cups of the photoeclectors were filled with water and detergent. We emptied the photoeclector traps weekly from the end of May until the beginning of July. Because we found other OSR pest species emerging from the ground in the exclusion experiment, we also determined their number. Specifically, we calculated the number of adult OSR pest weevils (*Ceutorhynchus* sp.) and adult cabbage stem flea beetles (*Psylliodes chrysocephala*).

### Sampling of ground-dwelling predators

In each OSR field, the activity density of ground-dwelling predators (Carabidae and Araneae) was measured with a pitfall trap placed 20 m distance from the exclusion experiment. All pitfall traps had a cup opening of 8 cm and were filled with a 1:3 ethylene glycol (automobile antifreeze, H. Kerndl GmbH)-water-mixture with detergent as trapping liquid^57^. The pitfall traps were protected by a small metal rooflet (25 cm × 25 cm and 15 cm high) to prevent flooding by rain. Crossed metal rods were placed over the cup opening to prevent small vertebrates from falling into the trap. All pitfall traps were set up on the 4^th^ of April and emptied fortnightly until the beginning of July when the whole experimental setting was removed. Overall, 126 samples were collected (18 sites × 7 collection intervals). We considered the abundance of carabid beetles, cursorial spiders and rove beetles as the total number of individuals for each sampling period per field.

### Statistical analyses

First, we tested the effect of the ‘exclusion’ treatment (factor: exclosure treatment versus open treatment), the density of pollen beetle larvae dropping to the ground and their interaction on the density of pollen beetle adults emerging from the ground using linear mixed effect models (LMMs) implemented in the R package ‘lme4’^58^. We also tested the effect of the ‘exclusion’ treatment on the density of adult OSR pest weevils emerging from the ground. Because the density of cabbage stem flea beetles was low, this group was not analysed. The site ID was included in the model as a random factor. Then, we tested the relative importance of parasitoids and ground-dwelling predators in controlling pollen beetle using the proportion of emerged pollen beetle adults (i.e. ratio between the density of pollen beetle adults emerging from the ground and the density of pollen beetle larvae dropping to the ground) as the response variable. The model included ‘parasitism rate’, ‘exclusion’ treatment and their interaction as fixed and ‘site ID’ as random effects.

In a further set of analyses, we investigated whether and to what extent the variation in the magnitude of the effect of parasitism and predation changed according to the proportion of non-crop habitats in the surrounding landscape. We used LMMs, with ‘site ID’ as a random factor, relating the density of emerged pollen beetle adults to the proportion of non-crop habitats, parasitism rate, ‘exclusion’ treatment and the two-way interactions between proportion of non-crop habitats and parasitism or ‘exclusion’ treatment. We also tested the effect of the proportion of non-crop habitats in the landscape on parasitism rate, densities of ground-dwelling predators (total, ground beetles, rove beetles and spiders) and pollen beetle larvae using LMMs with ‘sampling period ID’ as a random factor. We also repeated the analysis considering the proportion of forests and grasslands in the landscape, separately.

In all the models, we first built a full model with main and interactive terms and then simplified it by removing one-by-one the non-significant fixed terms, while respecting marginality. *F* and *P* values were interpreted using Satterthwaite’s approximations to determine denominator degrees of freedom in package ‘lmerTest’^59^. To improve normality and homoscedasticity of residuals, abundance data and proportion of emerged adult pollen beetles were ln-transformed, while parasitism rate was logit transformed. All analyses were conducted using R version 3.2.2^60^.

### Data availability

All data generated or analysed during this study are included in this published article (and its Supplementary Information files).

## Electronic supplementary material

Supplementary Information

Dataset S1
