# Supplementary material for: Complementarity among natural enemies enhances pest suppression
Source: Sci Rep. 2017 Aug 15;7:8172. doi: 10.1038/s41598-017-08316-z (PMC5557966; doi:10.1038/s41598-017-08316-z)
Supplement: Supplementary file 1 — Supplementary Information [file 41598_2017_8316_MOESM1_ESM.pdf]

Supplementary Information

Complementarity among natural enemies enhances pest suppression

Matteo Dainese, Gudrun Schneider, Jochen Krauss, Ingolf Steffan-Dewenter

This Supplementary Information contains the following information:

- Figure S1. Aerial imagery of the study area and the locations of the selected field sites and OSR fields.
- Figure S2. Relationship between ln-transformed number of adult pollen beetles emerging from the soil and ln-transformed number of pollen beetle larvae dropping to the ground (a), and effect of parasitism rate on the ln-transformed proportion of adult pollen beetles emerging from the soil (b).
- Figure S3. Relationship between parasitism rate and number of pollen beetle larvae dropping to the ground (a), and effect of the grassland cover (% of grasslands in a 1 km radius) on parasitism rate (b).
- Figure S4. Effect of landscape context on pest density.

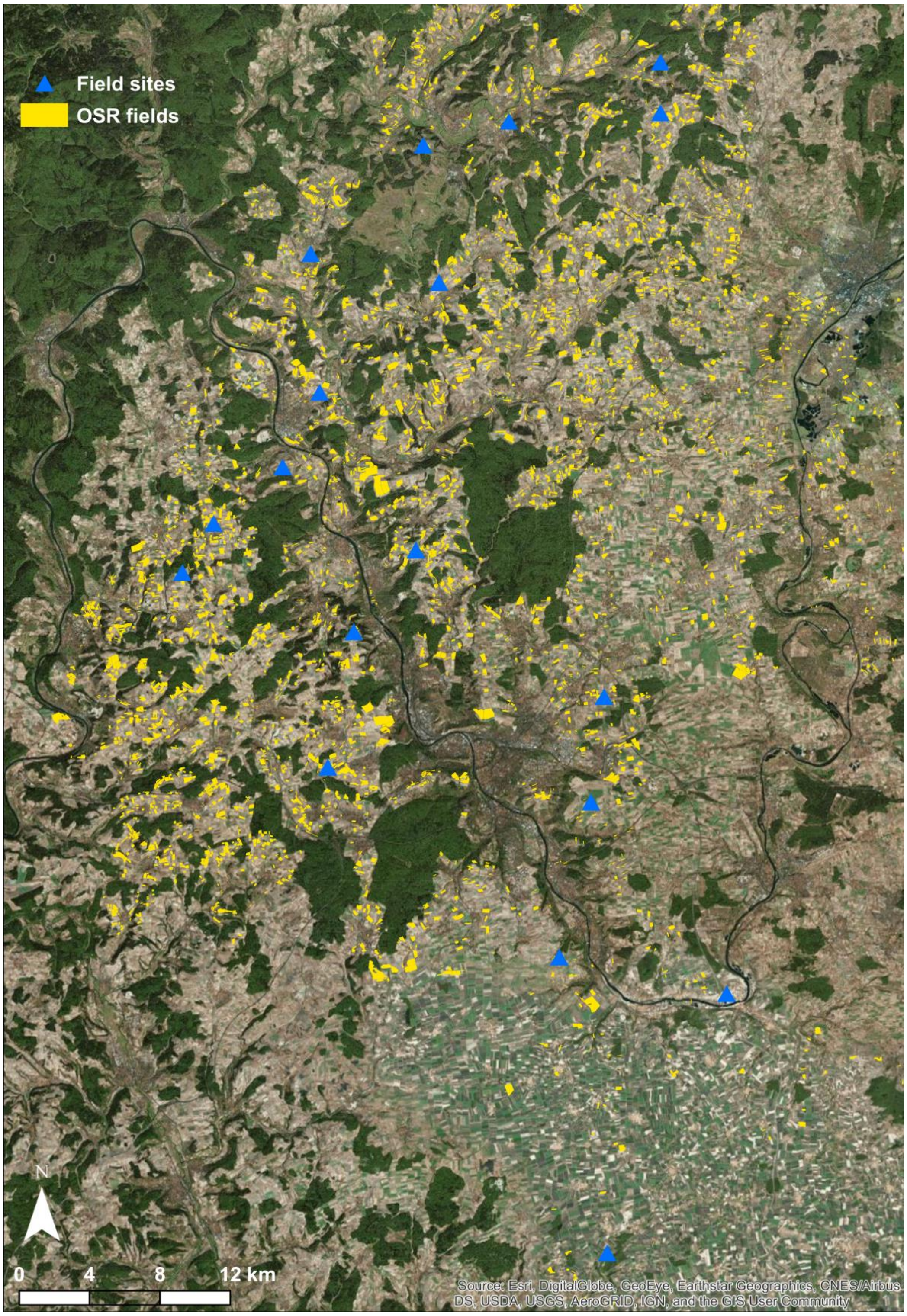

Figure S1. Aerial imagery of the study area and the locations of the selected field sites and OSR fields. Imagery from the ArcGIS World Imagery Basemap (Date of access: June 2017).

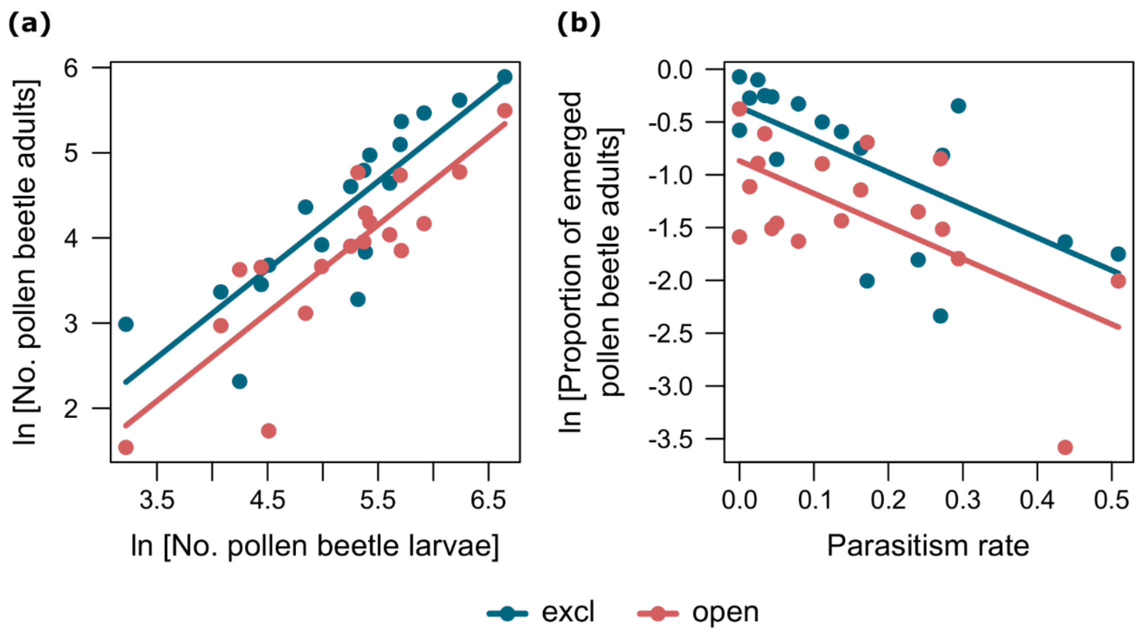

Figure S2. (a) Relationship between ln-transformed number of adult pollen beetles emerging from the soil and ln-transformed number of pollen beetle larvae dropping to the ground. (b) Effect of parasitism rate on the ln-transformed proportion of adult pollen beetles emerging from the soil. Separate relationships were reported for the two exclusion treatments (excl = exclosure treatment; open = open treatment). The interaction with treatment was found not significant ($P > 0.05$) in both models. Fitted lines are linear mixed model estimates from the model described in Table 1.

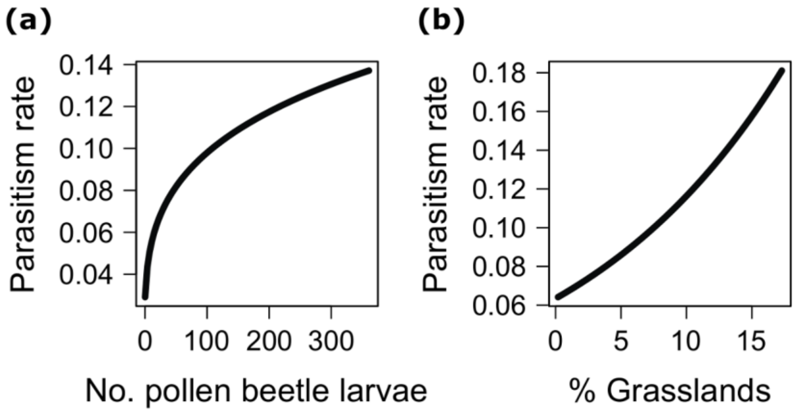

Figure S3. (a) Relationship between parasitism rate and number of pollen beetle larvae dropping to the ground. (b) Effect of the grassland cover (% of grasslands in a 1 km radius) on parasitism rate. Fitted lines are back-transformed linear mixed model estimates from the model described in Table 1.

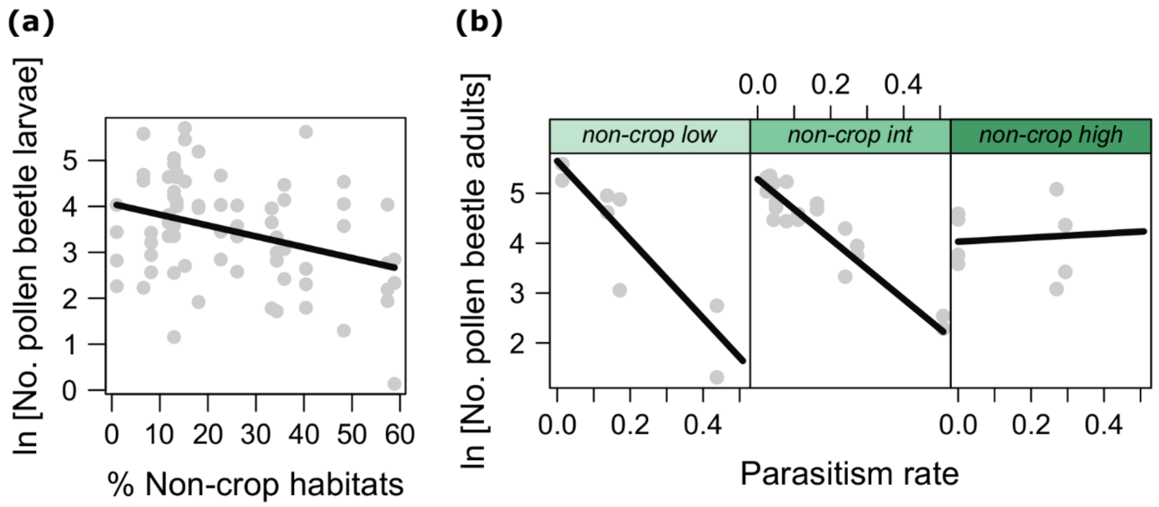

Figure S4. Effect of landscape context on pest density. (a) Effect of the proportion of non-crop habitats in the landscape on ln-transformed number of pollen beetle larvae dropping to the ground. (b) Interactive effect of parasitism rate and the proportion of non-crop habitats in the landscape on the number of adult pollen beetle emerging from the soil; panels are ranked from left to right according to increasing proportion of non-crop habitats (*non-crop low*, landscapes with low cover of non-crop habitats – 6%; *non-crop int*, landscapes with intermediate cover of non-crop habitats – 18%; *non-crop high*, landscapes with high cover of non-crop habitats – 50%). Fitted lines are linear mixed model estimates from the model described in Table 1.
